# Enhancing lycopene production in *Bacillus subtilis* by overcoming a critical enzymatic bottleneck

**DOI:** 10.3389/fbioe.2025.1670015

**Published:** 2025-08-29

**Authors:** Esha Rehman, Hawaibam Birla Singh, Minh Phuong Nguyen, Chonglong Wang, Sang-Hwal Yoon, Moonhyuk Kwon, Min-Kyoung Kang, Seon-Won Kim

**Affiliations:** ^1^ Anti-Aging Bio Cell Factory Regional Leading Research Center, Gyeongsang National University, Jinju, Republic of Korea; ^2^ Division of Applied Life Science (BK21 Four), Gyeongsang National University, Jinju, Republic of Korea; ^3^ School of Biology and Basic Medical Sciences, Soochow University, Suzhou, China; ^4^ Research Institute of Molecular Alchemy (RIMA), Gyeongsang National University, Jinju, Republic of Korea; ^5^ Plant Molecular Biology and Biotechnology Research Center, Gyeongsang National University, Jinju, Republic of Korea

**Keywords:** metabolic engineering, lycopene, *Bacillus subtilis*, MEP pathway, GGPP synthase

## Abstract

*Bacillus subtilis* a Generally Recognized As Safe (GRAS) microorganism, is an attractive chassis for producing high-value compounds in a safe and sustainable way. However, its potential for producing the C40 carotenoid lycopene has been limited by inefficient precursor supply and enzyme incompatibility. This study demonstrates that lycopene production in *B. subtilis* can be significantly enhanced through systematic metabolic engineering by rewiring the lycopene and methylerythritol phosphate (MEP) pathways. A synthetic lycopene biosynthesis pathway expressing the *crtE* gene from *Pantoea agglomerans*, which is commonly used for microbial lycopene production, failed to yield lycopene production in *B. subtilis*. However, replacing *crtE* with a multifunctional geranylgeranyl diphosphate synthase (GGPPS) from *Archaeoglobus fulgidus* successfully enabled lycopene synthesis. The optimization of the fermentation medium demonstrated that a combined carbon supply of glucose and glycerol markedly enhanced both cell growth and lycopene production in comparison with separate carbon sources. To further boost production, the methylerythritol phosphate (MEP) pathway was engineered by overexpressing the rate-limiting enzyme, 1-deoxy-D-xylulose-5-phosphate synthase (*dxs*), which resulted in a five-fold increase in lycopene titer after 72 h. Screening of various GGPPS enzymes revealed that *idsA* from *Corynebacterium glutamicum* was the most efficient, further increasing the yield. The final engineered strain achieved a lycopene titer of 55 mg/L in shake-flask cultivation, a significant improvement over the previously reported level in *B. subtilis*. These results demonstrate that targeted GGPPS selection and precursor pathway engineering are critical strategies for developing *B. subtilis* into a robust and sustainable platform for carotenoid production.

## 1 Introduction

*Bacillus subtilis* is a Gram-positive microorganism widely utilized in industrial biotechnology. Its GRAS (Generally Recognized As Safe) status and lack of endotoxin facilitate the purification of heterologous proteins and metabolites, making it a preferred host for food and pharmaceutical applications (Schallmey et al., 2004; Liu et al., 2013; Guan et al., 2015). Furthermore, *B. subtilis* exhibits broad substrate compatibility, resistance to harsh fermentation conditions, and an efficient protein secretion system, positioning it as an ideal chassis for sustainable biomanufacturing (Liu et al., 2023). Consequently, *B. subtilis* has shown promise as a chassis host for the synthesis of valuable compounds and drugs, such as squalene (Song et al., 2020), amorphadiene (Zhou et al., 2013), menaquinone-7 (Cui et al., 2019), riboflavin (Shi et al., 2009; Shi et al., 2014), and taxadiene (Abdallah et al., 2019).

Terpenoids, a large class of natural products, are synthesized from the universal C5 precursors isopentenyl diphosphate (IPP) and dimethylallyl diphosphate (DMAPP). While *Saccharomyces cerevisiae* and *Escherichia coli* have been the traditional hosts for terpenoid biosynthesis research, the potential of *B. subtilis* remains comparatively underexplored. Previous work in *B. subtilis* has focused on modifying the native 2-C-methyl-D-erythritol-4-phosphate (MEP) pathway. For instance, Xue and Ahring (Xue and Ahring, 2011) demonstrated that overexpressing *dxs* increased isoprene (C5) yield by 40%, whereas *dxr* overexpression had no effect. Early work demonstrated the feasibility of producing C30 carotenoids in *B. subtilis*. Yoshida et al. (2009) introduced the *crtM* and *crtN* genes from *Staphylococcus aureus* to synthesize these C30 compounds, which also conferred increased resistance to oxidative stress in the modified *B. subtilis* host. Building on this, (Xue et al., 2015) significantly enhanced C30 carotenoid yields by systematically overexpressing genes from the native MEP pathway. Strains engineered to co-express different subsets of MEP pathway genes along with the carotenoid genes achieved production levels more than 15-fold higher than strains harboring only *crtM* and *crtN*. For example, engineered strains containing gene cassettes such as Group A (*dxs*, *ispD*, *ispF*, and *ispH*) or Group B (*ispC*, *ispE*, *ispG*, and *ispA*) reached a C30 carotenoid yield of 9.1 mg/g dry cell weight.

Lycopene, a C40 tetraterpene carotenoid, is a potent antioxidant with widespread use in the food, pharmaceutical, and cosmetic industries (Siwach et al., 2018). It also serves as a key precursor for the synthesis of other valuable carotenoids. To date, the biotechnological production of lycopene has been extensively developed in non-GRAS organisms like *E. coli*, which raises safety and regulatory concerns for applications requiring high purity, such as in food and nutraceuticals. In this context, *B. subtilis* emerges as a superior alternative. As a GRAS organism, it provides an inherently safer platform, circumventing the endotoxin issues associated with Gram-negative hosts and simplifying downstream purification processes. Beyond its safety profile, *B. subtilis* is renowned for its robustness in large-scale industrial fermentations and its natural capacity for high-density growth, making it an industrially relevant chassis. However, despite these advantages, its potential for C_40_ carotenoid production has been underexplored and hindered by low titers of around 1.12 mg/L to 4.1 mg/L (Liu et al., 2023), often attributed to enzymatic incompatibilities and suboptimal precursor flux.

**FIGURE 1:**
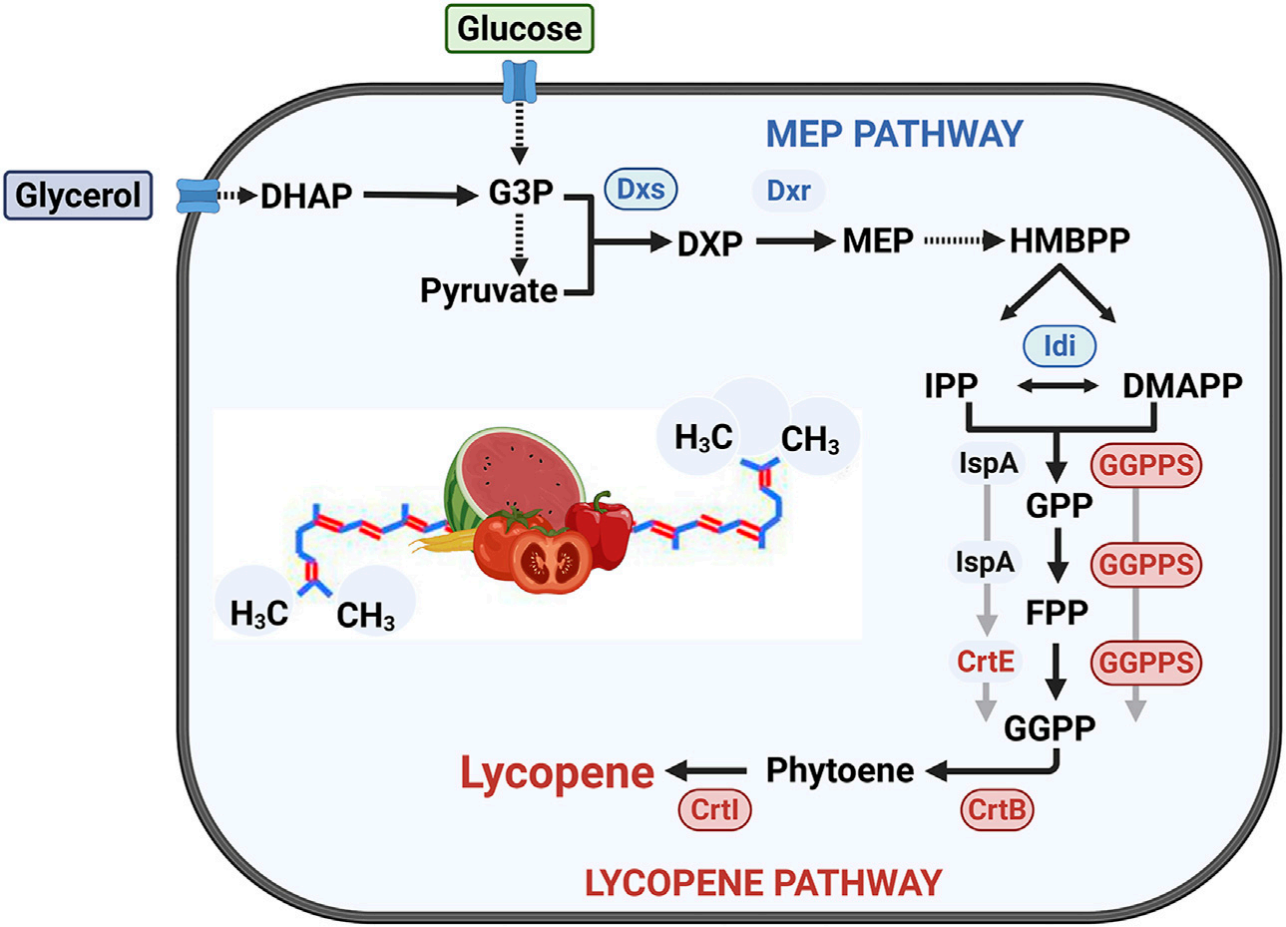
Metabolic pathway for lycopene biosynthesis in *B.* *subtilis* and corresponding engineering strategies. The native MEP pathway converts central metabolites derived from glucose and glycerol into the C5 precursors IPP and DMAPP. A heterologous pathway was then introduced to synthesize lycopene from these precursors. This study investigated two distinct routes for producing the key C20 intermediate, GGPP: (1) a route dependent on farnesyl diphosphate (FPP), utilizing the native FPP synthase (IspA) and the heterologous GGPP synthase (CrtE); and (2) a direct route bypassing FPP, which uses a multifunctional GGPP synthase (GGPPS) to convert C5/C10 precursors directly to GGPP. Key enzymes targeted for overexpression are encircled and highlighted. Abbreviations: (Enzymes) Dxs, 1-deoxy-D-xylulose-5-phosphate synthase; Dxr, 1-deoxy-D-xylulose-5-phosphate reductoisomerase; Idi, IPP isomerase; IspA, FPP synthase; CrtE, GGPP synthase; GGPPS, multifunctional GGPP synthase; CrtB, phytoene synthase; CrtI, phytoene desaturase. (Intermediates) G3P, glyceraldehyde-3-phosphate; DHAP, dihydroxyacetone phosphate; F6P, fructose-6-phosphate; DXP, 1-deoxy-D-xylulose 5-phosphate; MEP, 2-C-methyl-D-erythritol 4-phosphate; HMBPP, 1-hydroxy-2-methyl-2**(E)**-butenyl 4-pyrophosphate; IPP, isopentenyl diphosphate; DMAPP, dimethylallyl diphosphate; GPP, geranyl diphosphate; FPP, farnesyl diphosphate; GGPP, geranylgeranyl diphosphate.

This study aims to address this gap by systematically engineering *B. subtilis* for enhanced lycopene production. Our strategy involves constructing a functional heterologous pathway and optimizing precursor supply by targeting bottlenecks in the native MEP pathway (Figure 1). Specifically, we focus on screening for an efficient geranylgeranyl diphosphate synthase (GGPPS) and overexpressing key pathway genes, *dxs* and *idi*, to increase the metabolic flux towards lycopene. This work demonstrates the potential of *B. subtilis* as a viable and effective platform for producing lycopene and other high-value carotenoids.

## 2 Materials and methods

### 2.1 Bacterial strains, plasmids, and reagents

*E. coli* DH5α (F^−^φ80d*lacZ*M15 (*lacZYA* ^—^ *arg*F) U169 *deoR recA1 endA1 hsdR17* (rK^—^, mK^+^) *phoA supE44λ*¯ *thi*¯^1^ *gyrA96 relA1*) was used for cloning and plasmid propagation. *B. subtilis* 168 (ATCC 23857) was used as the host strain for lycopene production. Plasmids used are listed in Supplementary Table S1 and the full plasmid maps and annotated sequences for all key constructs are compiled into Supplementary_Data_Plasmid_Maps_And_Sequences. Restriction enzymes, T4 DNA Ligase (New England Biolabs), and Phusion DNA Polymerase (ThermoScientific, United States) were used for molecular cloning. HPLC-grade methanol was purchased from Daejung Chemical Co., Ltd. (South Korea).

### 2.2 Plasmids construction

Standard molecular biology techniques were employed. The lycopene biosynthesis operon (*crtE*, *crtB*, *crtI*, *ipiHP1*) was sourced from the plasmid pT-LYCm4 (Yoon et al., 2006). The genes encoding geranylgeranyl diphosphate synthase (GGPPS) were obtained from various sources: *gps* and *idsA* were amplified from the genomic DNA of *Archaeoglobus fulgidus* and *Corynebacterium glutamicum*, respectively, while *GGPPS1, GGPPS11* and *SlG1* were amplified from the cDNA of *Oryza sativa*, *Arabidopsis thaliana* and *Solanum lycopersicum*, respectively. The *dxs* and *dxr* genes were amplified from *B. subtilis* 168 genomic DNA. These genes were cloned into the BamHI/XbaI sites of the IPTG-inducible shuttle vector pHT100 using Gibson assembly. All constructs were verified by colony PCR, restriction mapping, and DNA sequencing (Cosmogenetech Co., Ltd, South Korea). Verified plasmids were transformed into *B. subtilis* 168 as previously described (Spizizen, 1958).

### 2.3 Culture media and conditions

Culture media were prepared using the medium composition described in the Difco manual (11th edition, Difco; BD Science, U.S.A). The reagents used for media preparation were purchased from BD Science (United States) and Sigma (United States). *E. coli* was cultured in 2 YT medium (16 g tryptone, 5 g sodium chloride, and 10 g yeast extract per 1 L) at 37 °C. *B. subtilis* seed cultures were grown in LB medium (10 g tryptone, 10 g sodium chloride, and 5 g yeast extract per 1 L). For lycopene production, a fermentation medium containing 5% (w/v) glucose, 5% (w/v) glycerol, 5% (w/v) soy peptone, and 0.06% (w/v) KH_2_PO_4_ was used. The fermentation medium was successfully used for the production of menaquinone-7 from *B. subtilis* (Sun et al., 2024). Antibiotics were added as required: ampicillin (100 μg/mL) for *E. coli* and chloramphenicol (5 μg/mL) for *B. subtilis*. Gene expression was induced with 1 mM IPTG. Seed cultures were grown at 37 °C, while main fermentations for lycopene production were conducted at 25 °C.

### 2.4 Shake-flask culture

Engineered *B. subtilis* strains were pre-cultured in 5 mL LB medium at 37 °C and 250 rpm for 8 h. The seed culture was then inoculated (2% v/v) into 20 mL of fermentation medium in a 300-mL baffled flask. The main culture was incubated at 25 °C and 180 rpm for up to 144 h. Samples were collected every 12 h for analysis. Cell growth was monitored by measuring optical density at 600 nm (OD_600_) (Beckman DU730, Germany). pH was measured with a pH meter B-212 (HORIBA, Japan).

### 2.5 Extraction and quantification of lycopene from *B. subtilis*

Lycopene was extracted from *B. subtilis* as described previously (Yoshida et al., 2009) with slight modifications. The 200 μL of culture broths were centrifuged at 12,000 rpm for 2 min to harvest cells, and the cell pellets were washed with 1 mL TE (10 mM Tris, 0.1 mM EDTA). The cells were centrifuged again for 1 min at 12,000 rpm, resuspended in 500 μL of TE and 250 μL of lysozyme (10 mg/mL), and incubated at 37 °C with shaking for 1 h. After 1 h of cell lysis, the cell lysates were centrifuged at 12,000 rpm for 1 min, and the pellets were then resuspended in 200 μL of methanol and 300 μL of dichloromethane (DCM) by vortexing and incubated at 55 °C for 15 min. Then, 300 μL of acetone was added, and the mixtures were incubated for an additional 15 min. After this, the samples were centrifuged for 10 min, and the supernatants were collected in HPLC vials. The extracts were used for HPLC analysis. All strains were cultured in triplicate under the same conditions, and each sample was extracted in parallel.

For quantification of Lycopene, lycopene standard (Sigma-Aldrich, United States; ≥90% purity) was used to prepare standard curve. Calibration curves were generated with *R* ^2^ > 0.998. For the lycopene analysis, standard solutions were prepared by dissolving 1 mg in 1 mL of acetone and 5 mg in 10 mL of acetone, respectively. Calibration curves were obtained using freshly prepared standard solutions in the range of 0.5–50 mg/L.

### 2.6 HPLC analysis of lycopene and residual carbon sources

Lycopene quantification was performed using a Shimadzu LC-20A HPLC system with Symmetry C18 (250 mm × 4.6 mm, 5 μm) and Sentry Guard C18 (15 mm × 4.6 mm, 5 μm) HPLC columns from Waters (Milford, United States). The mobile phase was a 70:30 mixture of methanol and acetonitrile at a flow rate of 1.5 mL/min, and lycopene was detected at 475 nm. The analysis was conducted at 40 °C. The chromatograms of both the standard and extracted samples are shown in Supplementary Figure S4.

For the analysis of residual glucose and glycerol, culture supernatants were collected by centrifugation and filtered (0.22 μm), and residual sugars were quantified using HPLC with refractive index detection with Aminex HPLC Columns from Bio-Rad.

## 3 Results

### 3.1 Construction of a functional lycopene pathway in *B. subtilis*

**FIGURE 2:**
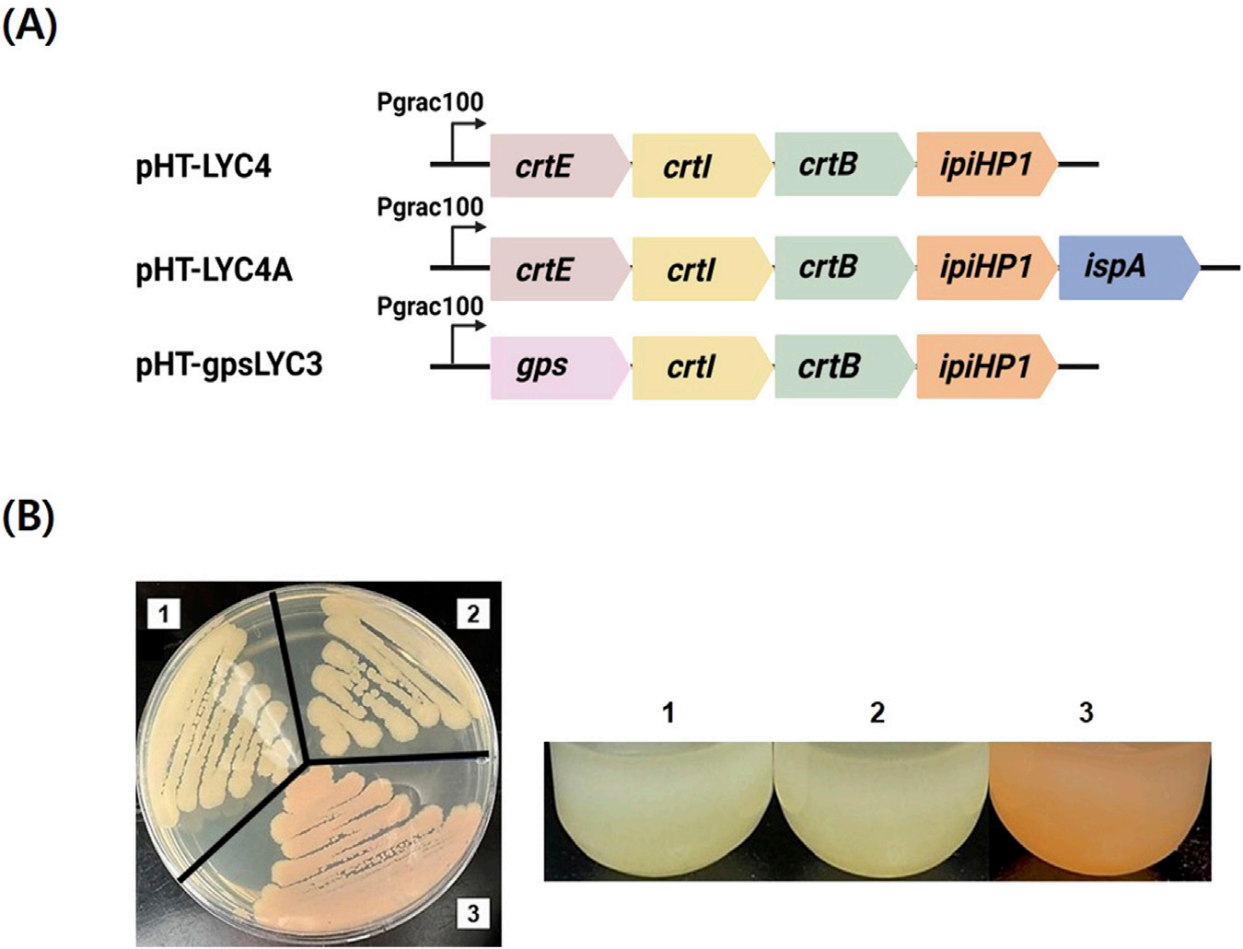
Initial construction of the lycopene biosynthesis pathway and resulting phenotypes. **(A)** Schematic representations of the key constructs used to establish the pathway. pHT-LYC4 contains the lycopene biosynthesis operon with *crtE* from *P. agglomerans*. pHT-LYC4A is a derivative of pHT-LYC4 with the additional overexpression of the native *ispA* gene. pHT-gpsLYC3 replaces the original *crtE* gene with the multifunctional *gps* gene from *A. fulgidus*. **(B)** Phenotypic analysis of *B. subtilis* colonies transformed with the corresponding plasmids. Strains harboring (1) pHT-LYC4 and (2) pHT-LYC4A exhibit no pigmentation, indicating a failure to produce lycopene. In contrast, the strain containing (3) pHT-gpsLYC3 displays a distinct pink-red color, providing visual confirmation of successful lycopene synthesis.

To enable lycopene production in *B. subtilis*, we first constructed the plasmid pHT-LYC4, an *E. coli* - *B. subtilis* shuttle vector expressing the lycopene biosynthesis genes (*crtE, crtB, crtI*) and the IPP isomerase gene (*ipiH*) (Figure 2A). As a crucial positive control, we first confirmed that this plasmid successfully produced lycopene in *E. coli*, resulting in a red-colored culture (data not shown). This demonstrated that the *crtEBI*-containing operon itself was functionally intact. However, when the same plasmid was introduced into *B. subtilis*, the resulting strain failed to produce any detectable lycopene, as indicated by the absence of the characteristic pink phenotype and further confirmed by HPLC analysis. This host-specific failure indicated that the bottleneck was not due to a faulty gene cassette but rather an issue within the *B. subtilis* metabolic context. We hypothesized two possibilities: 1) an insufficient supply of the precursor FPP, or 2) poor functionality of the CrtE enzyme within the *B. subtilis* host. To test the first hypothesis, we overexpressed the native FPP synthase gene, *ispA*, creating pHT-LYC4A. This modification also failed to yield lycopene, allowing us to largely exclude FPP availability as the primary limiting factor. By this deductive process, we identified the CrtE-catalyzed step as the critical host-specific bottleneck. To circumvent this issue, we pursued a strategy to bypass the FPP-dependent CrtE altogether by replacing it with *gps* from *A. fulgidus*, a multifunctional GGPPS. This new construct, pHT-gpsLYC3, successfully restored the pathway, leading to visible lycopene production (Figure 2B). This result confirmed that selecting a functionally compatible GGPPS is critical for establishing C40 carotenoid synthesis in *B. subtilis*. The potential of the multifunctional GGPP synthase to increase the flux towards lycopene production in *B. subtilis* was shown. Previous research has also shown that overexpression of *A. fulgidus gps* in *E. coli* dramatically boosted the production of astaxanthin (Wang et al., 1999).

### 3.2 Optimization of carbon source for lycopene production

**FIGURE 3:**
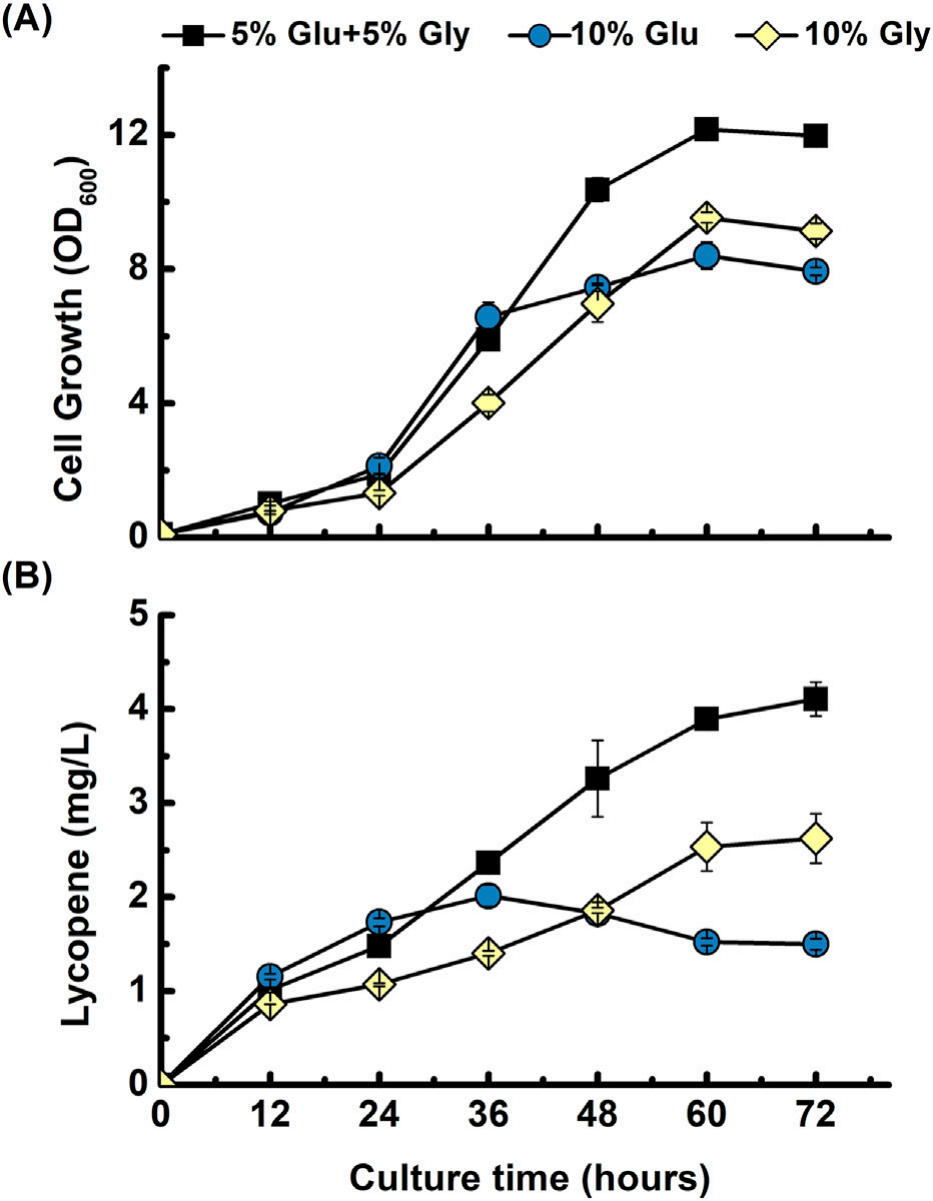
Comparison of different carbon sources for lycopene production. Time-course profiles of **(A)** cell growth (OD_600_), and **(B)** lycopene concentration (mg/L) are shown for the *B. subtilis* strain harboring the pHT-gpsLYC3 construct. The strain was cultivated in fermentation media containing three different carbon source compositions: a mixture of 5% glucose and 5% glycerol (■), 10% glucose alone (●), or 10% glycerol alone (♦). The data points represent the mean of three independent experiments, and error bars indicate the standard deviation.

To determine the optimal carbon source for lycopene production in *B. subtilis*, we evaluated cell growth and product synthesis using a fermentation medium containing three different carbon compositions. The base medium, successfully used for menaquinone-7 production in *B. subtilis* by Sun et al. (2024), contained a mixture of 5% glucose and 5% glycerol. To confirm the optimal carbon source for our lycopene engineered strain, a comparison was conducted against two single-source media containing either 10% glucose or 10% glycerol. Glycerol has been demonstrated to be a more effective carbon source for isoprenoid production than glucose in *E. coli*. In one study by Yoon et al. (2009) on *E. coli*, glycerol was found to yield the highest β-carotene production and cell growth among the various carbon sources tested. However, to determine whether same advantages would apply to *B. subtilis* comparison was conducted. As shown in Figures 3A,B, the carbon source composition significantly influenced both cell growth and lycopene production. In the medium with 10% glucose, cell growth and lycopene production patterns were initially comparable to those in the mixed-carbon medium for the first 36 h, after which both metrics stagnated. Conversely, the culture with 10% glycerol supported a slow but continuous increase in growth and production throughout the fermentation, though its final yields were lower than those of the mixed-carbon source. The medium containing both 5% glucose and 5% glycerol proved to be the most effective for *B. subtilis*, resulting in the highest final cell density and lycopene titer. The results obtained suggest that using this mixed carbon source is the most beneficial strategy to produce lycopene in our system. Therefore, this mixed-carbon strategy was employed for all subsequent experiments.

### 3.3 Overexpression of *dxs* significantly boosts lycopene production

**FIGURE 4:**
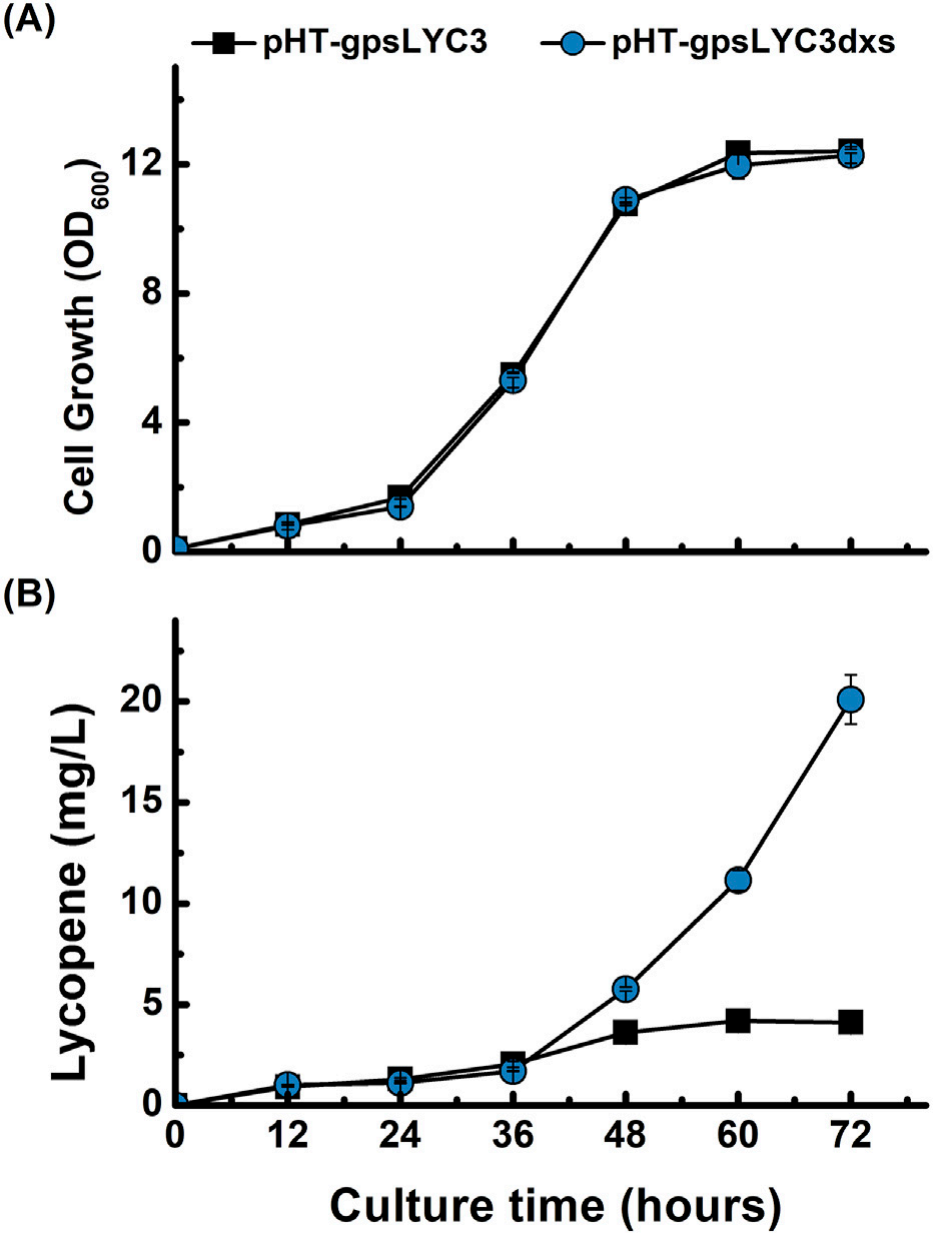
Effect of *dxs* overexpression on lycopene production and cell growth. Time-course profiles of **(A)** cell growth (OD_600_) and **(B)** lycopene concentration (mg/L). The performance of the control strain harboring pHT-gpsLYC3 (■) is compared with the strain overexpressing *dxs* from the plasmid pHT-gpsLYC3dxs (●). Data points represent the mean of three independent experiments, and error bars indicate the standard deviation.

The 1-deoxyxylulose-5-phosphate synthase encoded by *dxs* catalyzes the condensation of pyruvate and glyceraldehyde 3-phosphate to form 1-deoxyxylulose-5-phosphate (DXP) (Hess et al., 2013). To enhance the metabolic flux towards lycopene, we targeted the first committed step of the MEP pathway by overexpressing the rate-limiting gene *dxs*. A new plasmid, pHT-gpsLYC3dxs, was constructed and introduced into *B. subtilis*. Shake-flask cultivation revealed that the overexpression of *dxs* had no discernible negative effect on cell growth, as the growth profile was nearly identical to that of the control strain (Figure 4A). In contrast, lycopene production was significantly enhanced. The *dxs*-overexpressing strain exhibited a 2.6-fold increase in lycopene titer at 60 h and a substantial 5-fold increase at 72 h compared to the control (Figure 4B). This result clearly demonstrates that the availability of the precursor DXP is a major bottleneck and that upregulating *dxs* expression is a highly effective strategy for increasing lycopene yields in *B. subtilis*.

### 3.4 Screening identifies *idsA* as a superior GGPP synthase for lycopene production

**FIGURE 5:**
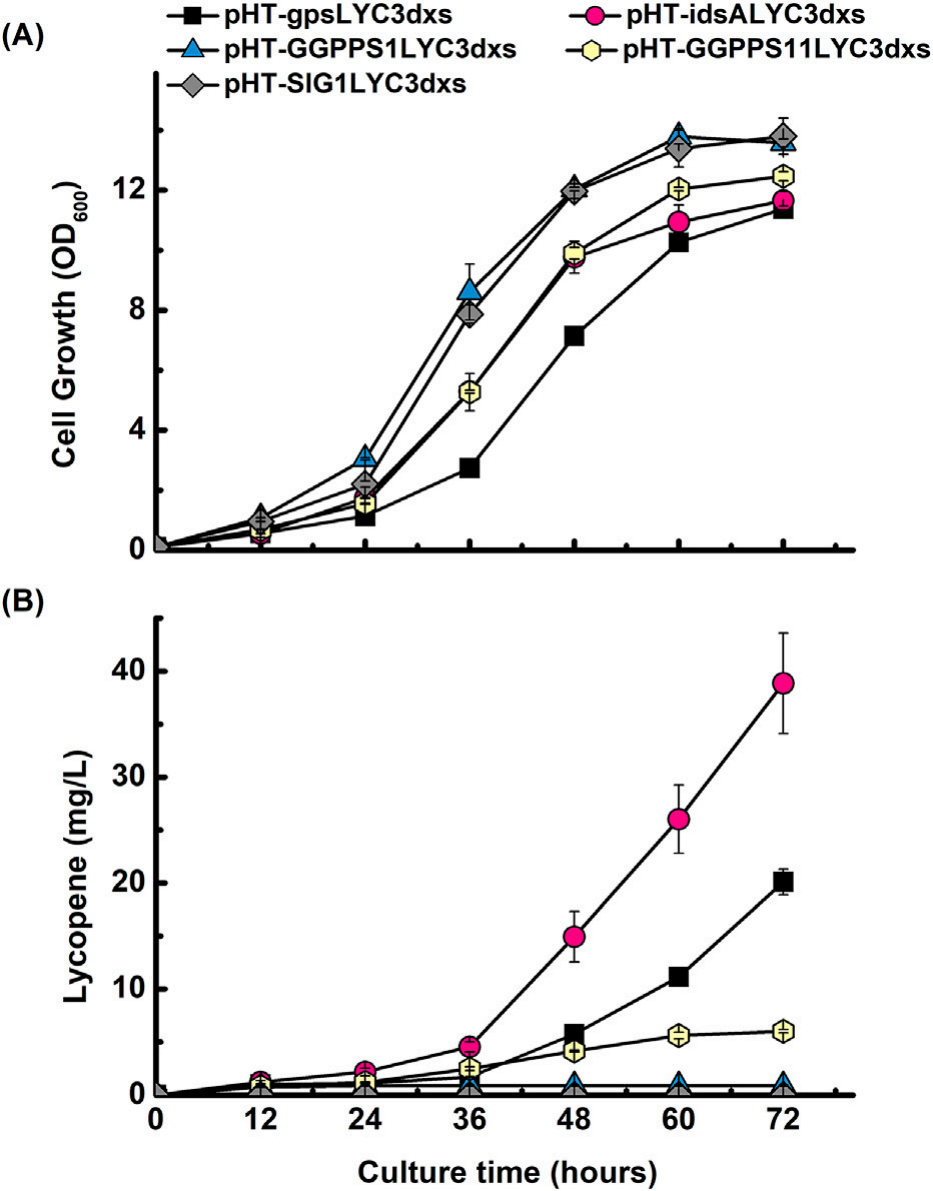
Screening of diverse GGPP synthases to optimize lycopene production. Time-course profiles of **(A)** cell growth (OD_600_) and **(B)** lycopene concentration (mg/L) for *B. subtilis* strains engineered to express different GGPP synthase enzymes. All constructs were built in the *dxs*-overexpressing background. The performance of five different synthases was compared: *gps* from *A. fulgidus* (■), *idsA* from *C. glutamicum* (●), *GGPPS1* from *O. sativa* (▲), *GGPPS11* from *A. thaliana* **(**●**)**, and *SlG1* from *S. lycopersicum* (♦). Data points represent the mean of three independent experiments, and error bars indicate the standard deviation.

To further optimize the pathway, we sought to identify a more efficient GGPP synthase than the *gps* from *A. fulgidus*. We hypothesized that enzymes from different evolutionary backgrounds might exhibit varied compatibility and performance within the *B. subtilis* metabolic environment. Therefore, we conducted a screening of a functionally and evolutionarily diverse panel of GGPPS enzymes to maximize the probability of identifying a superior candidate. The screening was conducted by replacing *gps* in the pHT-gpsLYC3dxs construct with four different candidate genes: *idsA* from *C. glutamicum*, *GGPPS1* from *O. sativa*, *GGPPS11* from *A. thaliana*, and *SlG1* from *S. lycopersicum* according to their known substrate specificities and varied evolutionary backgrounds (Supplementary Figure S1). Prokaryotic enzymes from *C. glutamicum* (a Gram-positive bacterium) and *A. fulgidus* (an archaeon) were chosen due to their established roles in native carotenoid biosynthesis pathways and reported efficiency in bacterial hosts, respectively. In contrast, enzymes from the eukaryotic plant sources *O. sativa*, *A. thaliana*, and *S. lycopersicum* were included to evaluate whether their documented broad substrate promiscuity might offer functional advantages in the *B. subtilis*. Shake-flask cultivation of these new strains revealed that while all exhibited similar cell growth profiles, their lycopene production levels varied significantly (Figures 5A,B). The strain expressing *idsA* from *C. glutamicum* demonstrated markedly superior performance, yielding the highest lycopene titer among all candidates. In contrast, the strain containing *SlG1* from *S. lycopersicum* failed to produce any detectable lycopene. A direct comparison showed that the *idsA* construct resulted in a ∼2.6-fold higher production than the gps construct at 60 h and a 1.9-fold increase at 72 h. This superior production, reaching approximately 40 mg/L, was visibly apparent from the deep red phenotype of the culture (Supplementary Figure S2). Consequently, the *idsA* enzyme was selected as the optimal GGPP synthase for all further engineering.

### 3.5 Overexpression of *dxr* fails to improve lycopene production

**FIGURE 6:**
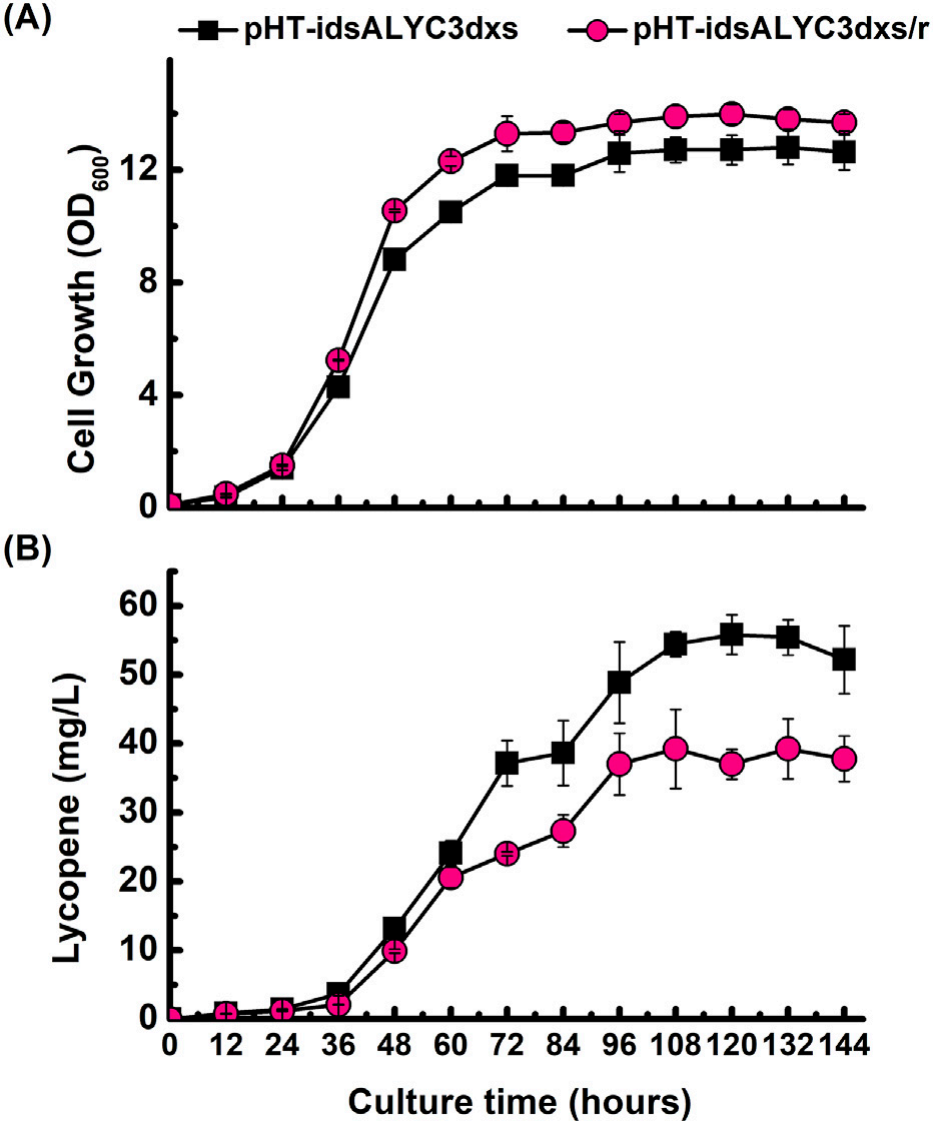
Effect of *dxr* co-overexpression on lycopene production. Time-course profiles of **(A)** cell growth (OD_600_) and **(B)** lycopene concentration (mg/L). The performance of the optimal strain harboring pHT-idsALYC3dxs (■) is compared with a derivative strain that additionally overexpresses the *dxr* gene, harboring pHT-idsALYC3dxs/r (●). Data points represent the mean of three independent experiments, and error bars indicate the standard deviation.

The second step of the MEP pathway involves the reduction and isomerization of DXP to produce MEP, which is carried out by *dxr*-encoded enzyme. It has been reported in *E. coli* that the overexpression of *dxr* along with *dxs* increased lycopene production (Kim and Keasling, 2001). To investigate the impact of modulating the second step of the MEP pathway, the *dxr* gene was overexpressed in our best-performing strain, creating the construct pHT-idsALYC3dxs/r. However, contrary to enhancing the pathway, the co-expression of *dxr* with *dxs* proved to be detrimental to lycopene synthesis. The final lycopene titer in the *dxr*-overexpressing strain was approximately 30% lower than that of the control strain lacking the additional *dxr* gene (Figures 6A,B). This result indicates that DXR is not a limiting factor for lycopene production in this engineered background, and its overexpression creates an imbalance that negatively affects the overall pathway flux.

Since the optimal strain (pHT-idsALYC3dxs) was still actively producing lycopene at the 72-h mark, the fermentation was extended to 144 h to determine the maximum achievable titer. Cell growth was observed to plateau after 96 h. Despite the cessation of growth, lycopene accumulation continued, increasing by an additional 1.4-fold between 72 and 144 h. This extended cultivation resulted in a final maximum lycopene production of 55 mg/L. Analysis of the culture supernatant at the end of the fermentation revealed the presence of residual glucose and glycerol (Supplementary Figure S3), indicating that the final production limit was not caused by carbon source depletion.

## 4 Discussion

This study successfully demonstrates the potential of *B. subtilis* as a robust platform for the enhanced production of lycopene. Through a systematic, multi-step engineering strategy, we achieved a final production of 55 mg/L, which represents an approximate 10-fold improvement over previously reported titers in this organism (Xu et al., 2023). This significant advancement was built on an alleviation of several key metabolic and enzymatic bottlenecks, providing valuable insights for future carotenoid engineering in *B. subtilis*. A critical finding of our work was the functional failure of the heterologous CrtE enzyme in *B. subtilis*, despite the same construct being functional in *E. coli*. This host-specific inactivity, even when the FPP precursor pool was supplemented via ispA overexpression, strongly suggests that the bottleneck is not a simple lack of substrate. Instead, we hypothesize that the CrtE enzyme is unable to effectively compete for the FPP substrate against potent native metabolic pathways in *B. subtilis*. Notably, *B. subtilis* is a prominent commercial producer of menaquinone-7 (MK-7), a pathway that also utilizes FPP as a key precursor (Cui et al., 2019). The strong intrinsic activity of this and other native pathways likely creates intense competition at the FPP node, preventing the foreign CrtE from efficiently channeling the flux towards GGPP. In contrast, the success of multifunctional synthases like *gps* and *idsA* can be attributed to their ability to bypass this competitive FPP branch point entirely. By directly synthesizing GGPP from earlier, less-contested precursors (IPP, DMAPP, and GPP), these enzymes circumvent the metabolic traffic jam at the FPP node. This highlights a crucial engineering principle for C40 carotenoid production in *B. subtilis*: the most effective strategy may not be to simply push a linear pathway, but to select enzymes that can navigate around the host’s entrenched metabolic highways.

Our results demonstrated that a mixed-carbon source of glucose and glycerol significantly outperformed single-source media for both cell growth and lycopene production. This synergistic effect can be attributed to several metabolic advantages in *B. subtilis*. Initially, the cells likely utilize glucose for rapid biomass accumulation, a phenomenon governed by Carbon Catabolite Repression (CCR). Once glucose is depleted, the cells switch to metabolizing glycerol. This metabolic shift is particularly beneficial for lycopene synthesis, as glycerol enters glycolysis as dihydroxyacetone phosphate (DHAP), providing a more direct route to glyceraldehyde-3-phosphate (G3P), a key precursor for the MEP pathway. This sequential utilization effectively separates the growth phase from the production phase, allowing for robust biomass generation followed by sustained product synthesis. This strategy avoids the early growth stagnation often seen with high glucose concentrations while leveraging glycerol’s efficient entry into the precursor pathway for terpenoid production.

Beyond establishing the core pathway, optimizing the supply of C5 precursors was essential. Our results reinforce that *dxs*, which catalyzes the first committed step of the MEP pathway, is the primary rate-limiting node for terpenoid synthesis in *B. subtilis*. Overexpression of *dxs* alone led to a notable five-fold increase in lycopene production without impairing cell growth, confirming that enhancing this initial flux is a highly effective strategy. In sharp contrast, further overexpression of *dxr*, the second pathway enzyme, was detrimental to production. This finding aligns perfectly with previous reports on isoprene synthesis in *B. subtilis* (Xue and Ahring, 2011) and clarifies that engineering the MEP pathway requires precise interventions; simply upregulating multiple enzymes can create metabolic imbalances that hinder overall productivity.

Finally, our fermentation analysis revealed that while the mixed-carbon source of glucose and glycerol was beneficial, production began to plateau after 96–120 h, even though the primary carbon sources were not depleted. This suggests that in the late stages of fermentation, lycopene synthesis becomes limited by factors other than carbon availability. These limitations could include the depletion of other essential nutrients (e.g., nitrogen, phosphorus), an insufficient supply of the reducing cofactor NADPH required for the MEP pathway, or potential feedback inhibition. Therefore, future optimization strategies should move beyond precursor supply and focus on these areas. Implementing dynamic gene expression systems, optimizing the supply of key nutrients and cofactors, and balancing the cellular redox state will be essential to extend the productive phase and push titers closer to commercially viable levels.

In conclusion, this study provides valuable strategic insights into the metabolic engineering of *B. subtilis* for C40 carotenoid production. While the individual methods employed, such as enzyme screening and pathway upregulation, are standard tools in the field, the key contribution of our work lies in elucidating a host-specific, logical engineering sequence for overcoming critical bottlenecks. We have demonstrated that for producing FPP-derived compounds in *B. subtilis*, a successful strategy involves two key steps: first, diagnosing and circumventing the intense substrate competition at the FPP node by selecting a multifunctional enzyme that bypasses it entirely; and second, after alleviating this primary enzymatic bottleneck, amplifying the precursor flux by targeting the correct rate-limiting step in the MEP pathway, which we confirmed to be *dxs*. This problem-solving approach, tailored to the unique metabolic context of *B. subtilis*, offers an effective engineering strategy that can be applied to enhance the production of other high-value terpenoids in this promising industrial chassis.

## 5 Conclusion

This study successfully engineered *B. subtilis* for enhanced lycopene production, achieving a final titer of 55 mg/L in shake-flask cultivation. By replacing an incompatible CrtE with a superior multifunctional GGPP synthase (*idsA*) and amplifying precursor flux via *dxs* overexpression, we systematically overcame key host-specific bottlenecks, resulting in a ten-fold improvement over previously reported levels in this organism. While the current titer is not yet industrially competitive and was achieved at the laboratory scale, this work establishes a critical foundation and a clear engineering strategy for developing *B. subtilis* as a safe and promising platform for carotenoid biosynthesis. Future efforts in process optimization and scale-up are required to unlock its full industrial potential.

## Data Availability

The original contributions presented in the study are included in the article/Supplementary Material, further inquiries can be directed to Esha Rehman: esharehman2000@gmail.com, Seon-Won Kim: swkim@gnu.ac.kr.
