# Supplementary material for: Enhancing lycopene production in Bacillus subtilis by overcoming a critical enzymatic bottleneck
Source: Front Bioeng Biotechnol. 2025 Aug 29;13:1670015. doi: 10.3389/fbioe.2025.1670015 (PMC12425915; doi:10.3389/fbioe.2025.1670015)

**Table S1. Plasmids used in this study**

| **Plasmid** | **Description** | **Sources** |
| --- | --- | --- |
| pT-LYCm4 | pTrc99A with *crtE, crtB,* and *crtI* from *P. agglomerans,* and *ipiHP1* from *H. pluvialis* | (Yoon et al., 2006) |
| pHT100 | *Bacillus subtilis - E. coli* shuttle vector, IPTG inducible P_grac100_, LacI, Amp^R^, Cmp^R^, | Lab stock |
| pHT-LYC4 | pHT100 harboring *crtEIB* from *P. agglomerans* and *ipiH* from *H. pluvialis* | This study |
| pHT-LYC4A | pHT100 harboring *crtEIB* from *P. agglomerans, ipiH* from *H. pluvialis* and *ispA* from *B. subtilis* | This study |
| pHT-gpsLYC3 | pHT100 harboring *gps* from *A. fulgidus, crtIB* from *P. agglomerans* and *ipiH* from *H. pluvialis* | This study |
| pHT-gpsLYC3dxs | pHT100 harboring *gps* from *A. fulgidus, crtIB* from *P. agglomerans,* *ipiH* from *H. pluvialis* and *dxs* from *B. subtilis* | This study |
| pHT-SlG1LYC3dxs | pHT100 harboring *SlG1* from *S. lycopersicum, crtIB* from *P. agglomerans,* *ipiH* from *H. pluvialis* and *dxs* from *B. subtilis* | This study |
| pHT-GGPPS1LYC3dxs | pHT100 harboring *OsGGPPS1* from *O. sativa, crtIB* from *P. agglomerans,* *ipiH* from *H. pluvialis* and *dxs* from *B. subtilis* | This study |
| pHT-GGPPS11LYC3dxs | pHT100 harboring *AtGGPPS11* from *A. thaliana, crtIB* from *P. agglomerans,* *ipiH* from *H. pluvialis* and *dxs* from *B. subtilis* | This study |
| pHT-idsALYC3dxs | pHT100 harboring *idsA* from *C. glutamicum, crtIB* from *Pantoea agglomerans,* *ipiH* from *Haematococcus pluvialis* and *dxs* from *B. subtilis* | This study |
| pHT-idsALYC3dxs/r | pHT100 harboring *idsA* from *C. glutamicum, crtIB* from *P. agglomerans,* *ipiH* from *H. pluvialis, dxs* and *dxr* from *B. subtilis* | This study |

**Table S2. GGPP Synthases screened in this study**

| **Gene Name (Abbreviation)** | **Organism** | **Substrate Specificity** | **Gene ID** |
| --- | --- | --- | --- |
| AfGGPPS (*gps*) | *Archaeoglobus fulgidus* | IPP, DMAPP, GPP, FPP | WP_048064551 |
| CgGGPPS (i*dsA*) | *Corynebacterium glutamicum* | IPP, DMAPP, GPP, FPP | G18NG-11764 |
| OsGGPPS (*GGPPS1*) | *Oryza sativa* | IPP, DMAPP, GPP, FPP | XM_015792500.3 |
| AtGGPPS (*GGPPS11*) | *Arabidopsis thaliana* | IPP, DMAPP, GPP, FPP | At4g36810 |
| SlGGPPS (*SlG1*) | *Solanum lycopersicum* | IPP, GPP, FPP | Solyc11g011240 |

**Figure S1. Schematic of the plasmid constructs used for GGPP synthase screening.** All constructs share a common genetic backbone consisting of the lycopene synthesis genes (*crtI* and *crtB* from *P. agglomerans*), the IPP isomerase (*ipiHP1* from *H. pluvialis*), and the MEP pathway gene (*dxs* from *B. subtilis*). The constructs differ only in the geranylgeranyl diphosphate synthase (GGPPS) gene used. The five GGPPS enzymes screened were: *gps* from *Archaeoglobus fulgidus*, *idsA* from *Corynebacterium glutamicum*, *GGPPS1* from *Oryza sativa*, *GGPPS11* from *Arabidopsis thaliana*, and *SlG1* from *Solanum lycopersicum*.


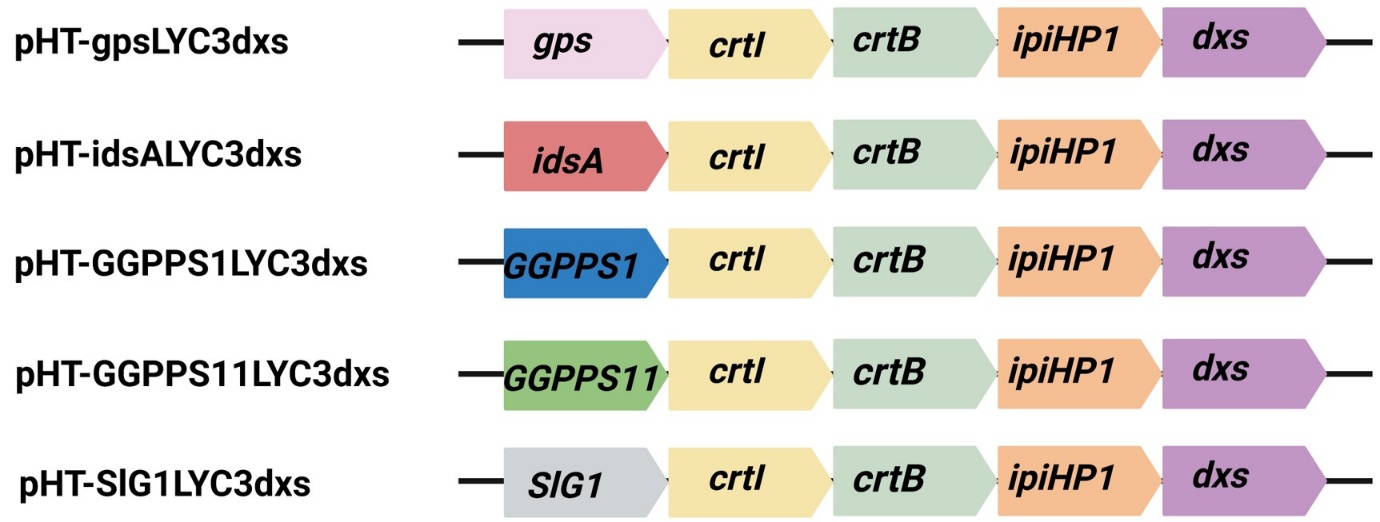


**Figure S2. Time-course visualization of lycopene production in the optimal strain.** The images display the color phenotype of the *B. subtilis* strain harboring pHT-idsALYC3dxs, which was identified as the most effective construct. The culture broth at 12-hour intervals shows a progressive color change from a pale to a deep red by 72 hours. The photograph on the right shows the final liquid culture at 72 hours. This deepening pigmentation provides a clear visual confirmation of the high-level accumulation of lycopene over the course of the fermentation.


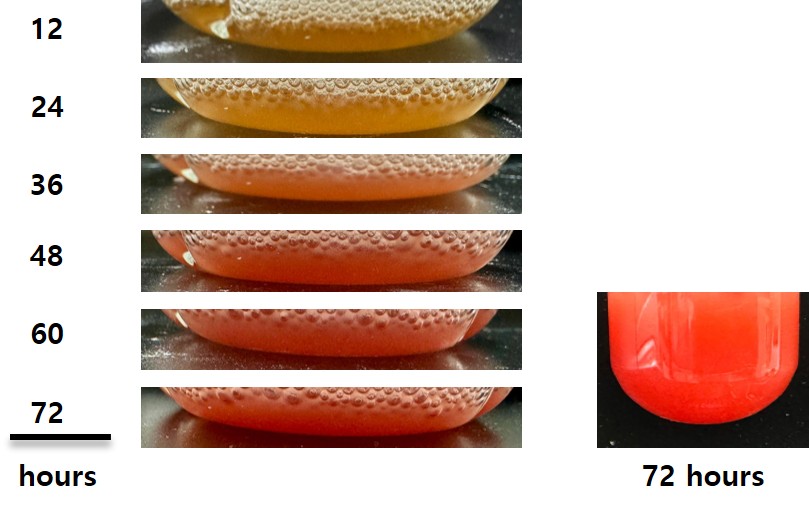


**Figure S3. Analysis of residual carbon sources during extended fermentation.** The graph displays the concentration of residual glucose (closed symbols) and glycerol (open symbols) remaining in the culture medium over the 144-hour fermentation period. The analysis was conducted for two strains: the optimal lycopene-producing strain (pHT-idsALYC3dxs) and the dxr-overexpressing strain (pHT-idsALYC3dxs/r). The data show that neither glucose nor glycerol was fully depleted by the end of the cultivation period for either strain.


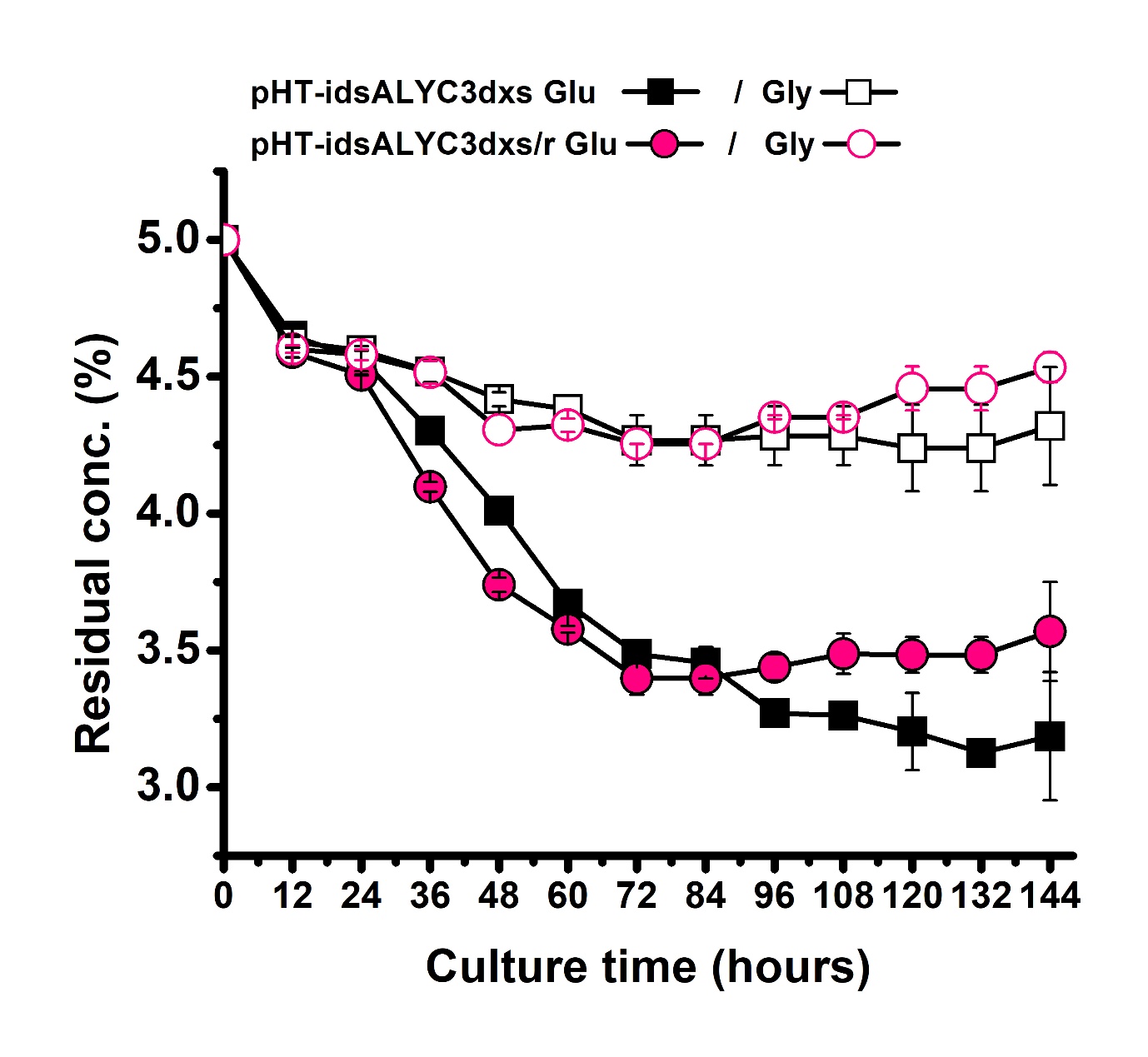


**Figure S4. Representative HPLC chromatograms:** Lycopene standard (black) and lycopene extracted from engineered *Bacillus subtilis* culture (red). Lycopene was detected at 475 nm. The retention time of the sample peak matched that of the standard (~18.5 min), confirming compound identity.


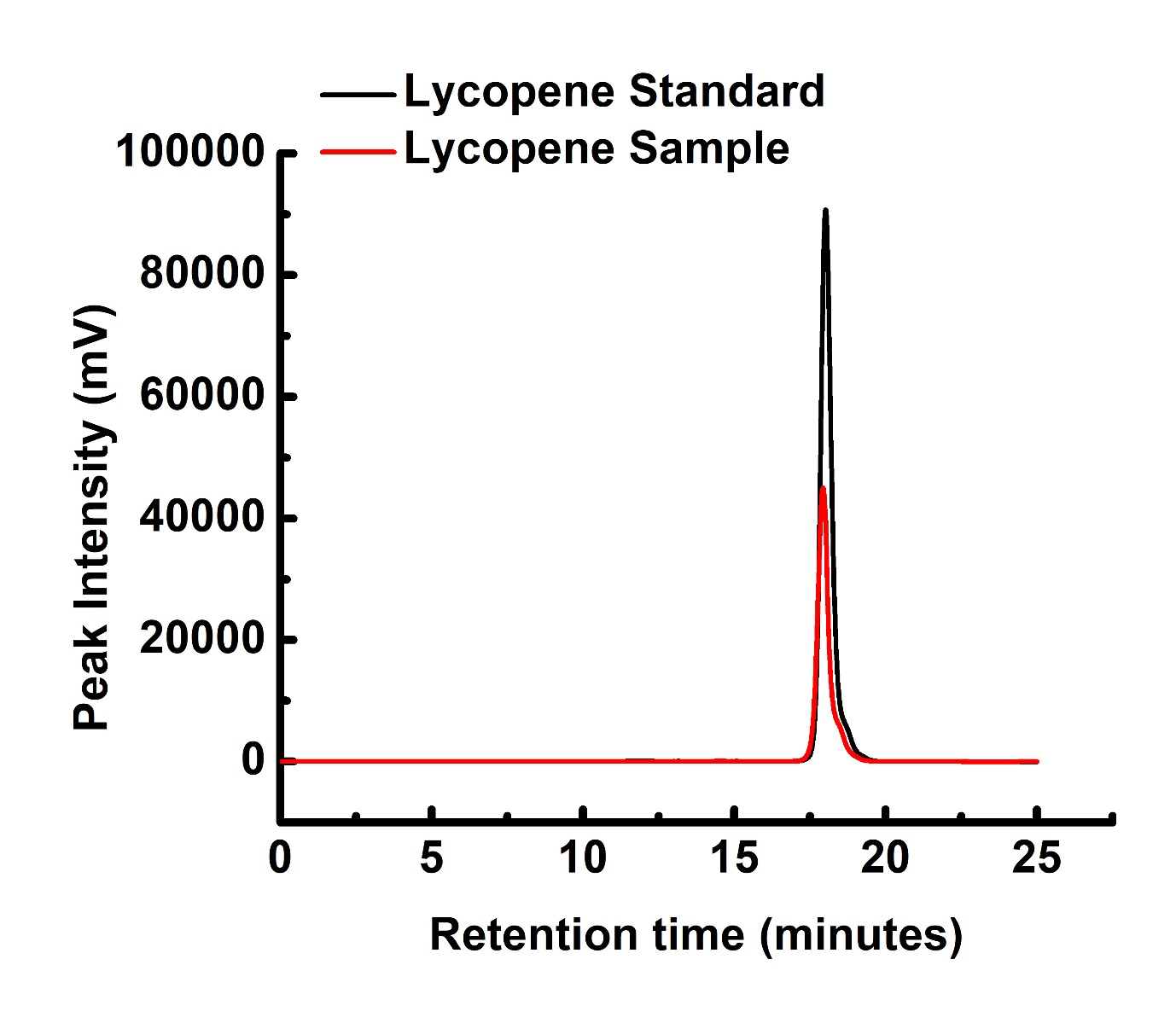

Supplement: Supplementary file 1 [file Supplementaryfile1.docx]
